# Development of Large-Scale Stopped-Flow Technique and its Application in Elucidation of Initial Ziegler–Natta Olefin Polymerization Kinetics

**DOI:** 10.3390/polym11061012

**Published:** 2019-06-07

**Authors:** Ashutosh Thakur, Toru Wada, Patchanee Chammingkwan, Minoru Terano, Toshiaki Taniike

**Affiliations:** Graduate School of Advanced Science and Technology, Japan Advanced Institute of Science and Technology, 1-1 Asahidai, Nomi, Ishikawa 923-1292, Japan; thakur@jaist.ac.jp (A.T.); toruwada@jaist.ac.jp (T.W.); chamming@jaist.ac.jp (P.C.); terano@jaist.ac.jp (M.T.)

**Keywords:** Ziegler–Natta catalysis, active sites, propylene polymerization, kinetics, stopped-flow technique

## Abstract

The stopped-flow (SF) technique has been extensively applied to study Ziegler–Natta (ZN) olefin polymerization kinetics within an extremely short period (typically <0.2 s) for understanding the nature of the active sites as well as the polymerization mechanisms through microstructure analyses of obtained polymers. In spite of its great applicability, a small amount of polymer that is yielded in a short-time polymerization has been a major bottleneck for polymer characterizations. In order to overcome this limitation, a large-scale SF (LSF) system has been developed, which offers stable and scalable polymerization over an expanded time range from a few tens milliseconds to several seconds. The scalability of the LSF technique has been further improved by introducing a new quenching protocol. With these advantages, the LSF technique has been effectively applied to address several unknown issues in ZN catalysis, such as the role of physical and chemical transformations of a catalyst on the initial polymerization kinetics, and regiochemistry of ZN propylene polymerization. Here, we review the development of the LSF technique and recent efforts for understanding heterogeneous ZN olefin polymerization catalysis with this new system.

## 1. Introduction

Since their invention, heterogeneous Ziegler–Natta (ZN) catalysts have been in the forefront of the catalyst technology of industrial polyolefin production, especially isotactic polypropylene (PP) [1]. ZN pro-catalysts typically contain multiple types of Ti species formed on surfaces of activated MgCl_2_ by the adsorption of TiCl_4_, together with a Lewis base (internal donor) to improve catalyst stereospecificity [2,3,4]. The hierarchical agglomeration of primary building units of TiCl_4_/internal donor/MgCl_2_ leads to the formation of multi-grained catalyst particles having a broad range of pores. For polymerization, these pro-catalysts are activated with an alkylaluminum cocatalyst in the presence of an additional Lewis base called as external donor. Thus, ZN catalysts possess complicated chemical and structural features over multiple length scales [5,6,7]. These complexities, on the other hand, positively contribute to the remarkable commercial success of ZN catalysts in the industrial polyolefin manufacturing. For instance, their multi-site nature results in polymers with broad molecular weight distributions, which are suitable for extrusion and injection molding applications [8,9]. Also, the multi-grained catalyst particles with hierarchical porous structures offer long standing catalytic activity as well as morphology control of the final polymer particles for efficient plant operation through the fragmentation and replication phenomena [10]. In spite of these advantages, the heterogeneous nature and scarce concentration of the active species formed on solid support make it extremely challenging to characterize the active sites using spectroscopic techniques [11]. In addition, the active sites of ZN catalysts are transient in nature due to facile formation/deactivation/transformation during polymerization, and as a result, microstructural characterizations of the product polymers are not straightforward for fingerprinting the nature/states of the active sites in order to understand several aspects of the polymerization mechanisms. At the industrial level, this catalyst system has been continuously developed in terms of empirical performance improvement. However, progress in regard to a deeper understanding of the fundamental aspects of ZN catalysis is quite slow, mainly due to the above-mentioned limitations.

Investigation of reaction kinetics is required for basic understanding of any catalysis. In the case of ZN catalysis, elucidation of polymerization kinetics through the determination of kinetic parameters, such as the average propagation rate constant (*k*_p_) and active site proportion per Ti represented by the active-site concentration ([C*]), is acknowledged as a challenging task. This is because of the time-dependent variation of the kinetic parameters due to a series of chemical and physical phenomena, those occur during polymerization. To address this issue, Terano and Keii invented a stopped-flow (SF) method, by which quasi-living olefin polymerization can be performed within an extremely short period (typically <0.2 s), which is less than the lifetime of the growing polymer chains [12,13,14]. The implication of the method was based on the fact that in ZN catalysis, a number of important reactions take place within a fraction of seconds such as the activation of supported Ti species on the exposed surfaces by an alkylaluminum, chain propagation, chain transfer, etc. Thus, a time-resolved investigation of an early period of polymerization by the SF method enables us to derive the kinetic parameters, which account for the intrinsic features of the active sites just after their formation and the polymerization mechanisms through the analyses of polymer microstructures [15].

**Figure 1:**
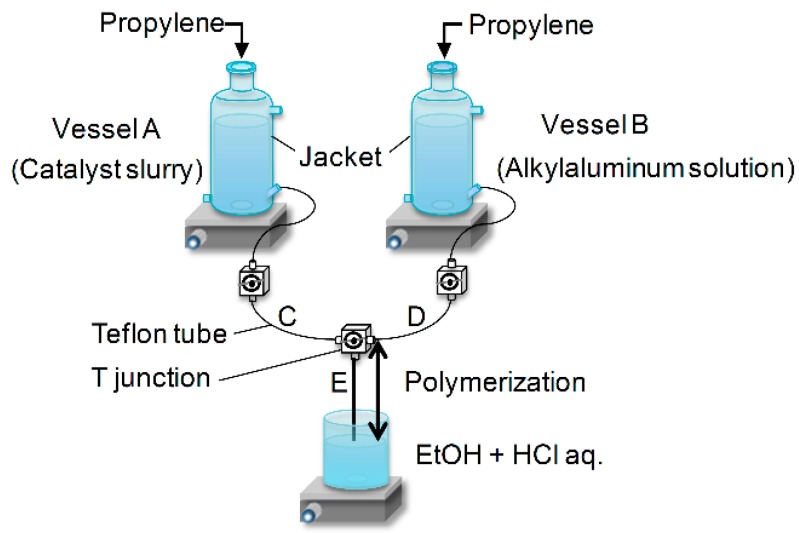
Conventional two-vessel stopped-flow (SF) apparatus [21].

The original two-vessel SF apparatus is illustrated in Figure 1. Two specially designed glass vessels A and B equipped with a jacket and a magnetic stirrer are charged with a catalyst slurry and an alkylaluminum solution. After saturation with olefin monomer, the catalyst slurry and alkylaluminum solution are simultaneously flown out through the Teflon tubes C and D by pressuring the vessels. Polymerization starts at the mixing point T, continues in the Teflon tube E and is finally quenched at the tube end by casting the polymerization slurry into an excess amount of acidic ethanol. Because of instantaneous catalyst activation at the T junction, as well as instantaneous quenching at the tube end, the polymerization time can be precisely controlled in the range of 0.05–1.0 s only by changing the length of the tube E. Owing to this feature, the SF polymerization has been recognized as a more reliable technique to determine the kinetic parameters in olefin polymerization than alternative techniques such as the ^14^CO/RO^3^H radio tagging method [16,17], where undesired multiple insertions of ^14^CO or nonselective insertions of RO^3^H in alkylaluminum-terminated dead chains often lead to the overestimation of [C*]. However, it should be noted that the typical SF polymerization is performed under model conditions, such as at low temperatures and low monomer pressure. Thus, the information derived from SF polymerization is more likely for industrial prepolymerization, which is performed under mild conditions to control the initial particle morphology and polymerization kinetics [18]. More realistic polymerization can be performed using a high-pressure SF apparatus in the quasi-living stage [19,20], while its elaboration is beyond the scope of the present review.

**Figure 2:**
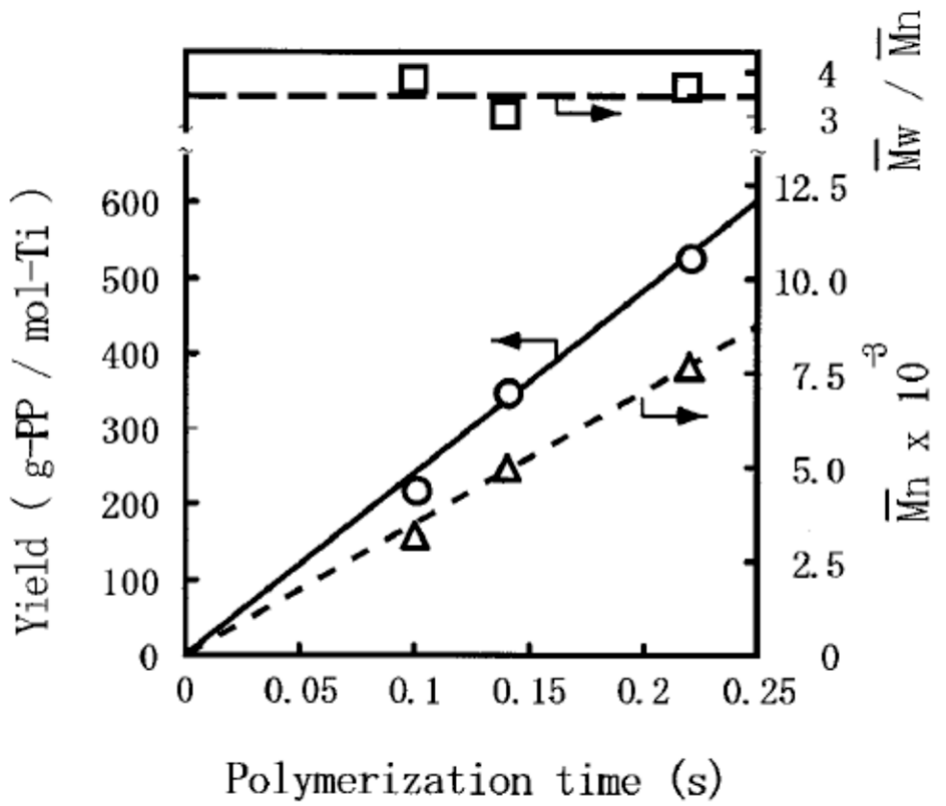
Yield, *M*_n_ and *M*_w_/*M*_n_ development in SF propylene polymerization using a TiCl_4_/ethylbenzoate/MgCl_2_-based catalyst [22].

Since active site transformation and deactivation are negligible at an early stage of olefin polymerization, the kinetic parameters *k*_p_, [C*], and the chain transfer rate constant (*k*_tr_) can be determined in a time-independent manner by the following equations [22],

(1) $$Y=k_{p}\left[M\right]\left[C^{*}\right]t$$

(2) $$M_{n}=M_{0}\frac{k_{p}\left[M\right]\left[C^{*}\right]t}{\left[C^{*}\right]+k_{tr}\left[C^{*}\right]t}$$

where *Y*, [M], *t*, *M*_n_, and *M*_0_ correspond to the polymer yield, monomer concentration in the polymerization medium, polymerization time, number-average molecular weight of polymer, and molar mass of monomer, respectively. In quasi-living polymerization process, the polymer yield and *M*_n_ develop linearly with the time (Figure 2) through the origin [22], suggesting the instantaneous activation of the catalyst (i.e., no induction period) as well as negligible occurrence of chain-transfer reactions (*k*_tr_ ≈ 0). Therefore, Equation (2) simplifies to

(3) $$M_{n}=M_{0}k_{p}\left[M\right]t$$

Since *M*_0_ and [M] are known parameters, *k*_p_ can be determined from the linear plot of *M*_n_ against *t* based on Equation (3).

Thus, the SF technique has been proven to be a powerful tool for the precise determination of the kinetic parameters *k*_p_ and [C*]. In addition, Terano et al. have applied the SF method to study propylene polymerization kinetics in combination with fingerprint techniques based on ^13^C-NMR spectroscopy and temperature rising elution fractionation (TREF) to associate the kinetic and stereospecific features of individual types of active sites [23,24,25,26]. Most notably, they clarified that [22,27,28,29,30,31,32]:

i) The broad molecular weight distribution of polyolefins produced by ZN catalysts is mainly due to the active site heterogeneity;
ii) The formation of active sites represented by [C*] is directly related to the reactivity of an employed alkylaluminum cocatalyst;
iii) The intrinsic nature of the active sites represented by *k*_p_ is closely associated with the stereospecificity of the active sites, while it is hardly affected by catalyst fragmentation, the type and concentration of alkylaluminum cocatalysts as well as catalyst preparation method;
iv) Interaction between a catalyst and a cocatalyst is plausibly an important factor for the formation of active sites with the highest isospecificity;
v) Activity enhancement in the copolymerization of propylene with ethylene at the quasi-living stage is attributed to *k*_p_ enhancement, plausibly due to the reactivation of dormant sites by more reactive ethylene or faster insertion of propylene after the insertion of less bulky ethylene. In the later stage of the polymerization, accelerated fragmentation of catalyst particles and faster diffusion of monomers induced by less crystalline copolymer likely contribute to the activity enhancement.

They also exploited the quasi-living polymerization feature of the SF technique to fabricate real polypropylene-block-poly(propylene-co-ethylene) copolymer with ZN catalysts using a modified three-vessel SF apparatus [33,34]. These types of block copolymers have been exploited as an effective compatibilizer in the melt-blending of PP with polyethylene (PE) [35]. In addition, the role of catalyst macropores to influence the early fragmentation process was confirmed by imaging the polymer/catalyst morphology development in the initial stage of the SF propylene polymerization [36,37].

In spite of the great applicability of the SF technique in studying heterogeneous ZN catalysts and their catalysis, a small amount of obtained polymer in a short polymerization time is problematic for precise ^13^C-NMR analysis as well as for other characterizations. Moreover, when the polymerization is performed beyond 1.0 s, the technique suffers from inefficient flow of reaction slurry that is caused by a larger amount of polymer formation inside the Teflon tube. In order to alleviate these problems, Taniike et al. developed a large-scale stopped-flow (LSF) system based on the identical principle of the conventional SF technique but with a greatly differed system design [21]. The new system offers improved flow stability for sufficiently elongated polymerization (up to several seconds) and the volume scalability to increase the polymer yield per batch. The scalability of the LSF polymerization was further improved by introducing a new quenching protocol [38], which made it possible to perform adequate characterizations even for the polymers obtained in ultra-short-time (<0.1 s) polymerization. Most importantly, the expansion in the accessible range of the polymerization time from a few tens of milliseconds to several seconds, the LSF technique has made it feasible to understand the chemical and physical transformations of a ZN catalyst occurring at the early stage of the polymerization [39], as well as the dormant processes in ZN propylene polymerization. This review summarizes the development of the LSF technique and the recent progress in understanding ZN propylene polymerization catalysis with this new system.

## 2. Development of Large-Scale Stopped-Flow Technique

**Figure 3:**
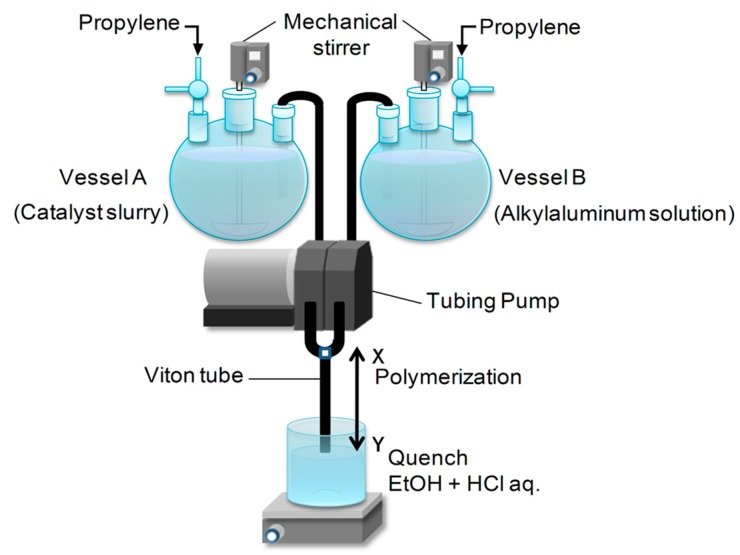
Two-vessel large-scale stopped-flow (LSF) apparatus [21].

In order improve the flow stability and the volume scalability, the LSF technique was developed [21]. The LSF apparatus is illustrated in Figure 3. A catalyst slurry and an alkylaluminum solution saturated by monomer were placed in two commercially available 2 L three-neck flasks (A and B), which were then immersed in a circulating water bath. To maintain the homogeneous slurry concentration inside the catalyst flask, the magnetic stirrer of the conventional apparatus was replaced by a mechanical stirrer. Stable transfer of the catalyst slurry and alkylaluminum solution was ensured using a tubing pump, operating between 0 to 600 rmp, and chemically inert Viton tubes (typical internal diameter of 3.1 mm). Finally, instantaneous quenching was achieved by casting the polymerization slurry into an excess amount of acidic ethanol, which was vigorously stirred using a homogenizer. The polymerization time can be precisely adjusted through either the flow rate or the length of the tube between X and Y.

**Figure 4:**
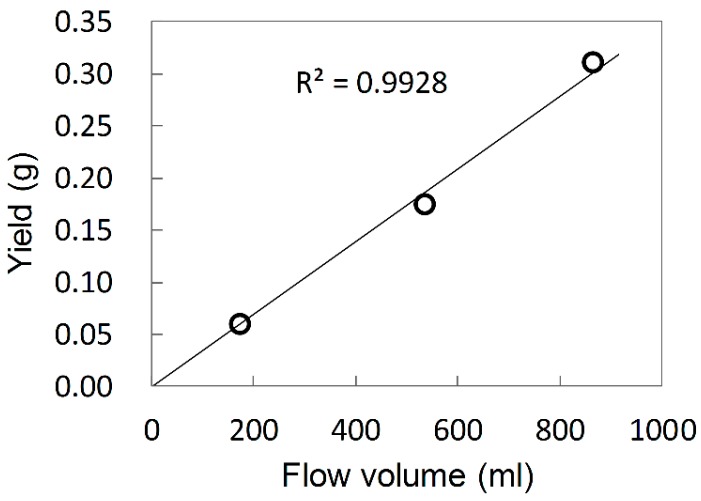
Scalability test in LSF propylene polymerization. The tube length was fixed at 0.15 s, while the cumulated flow volume was increased [21].

**Figure 5:**
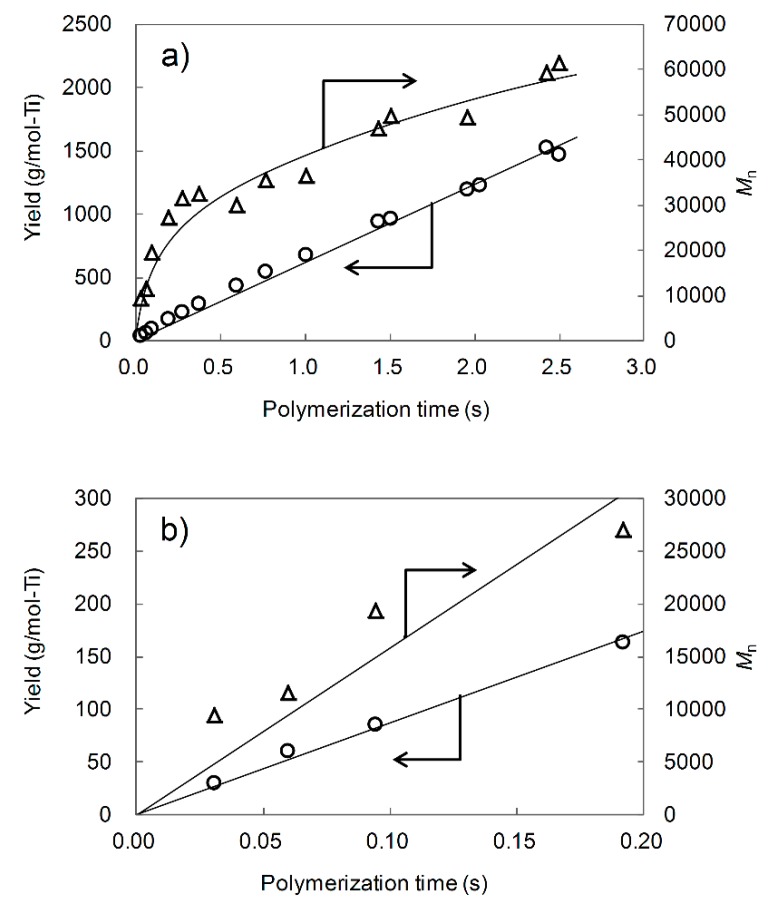
(**a**) Yield and *M*_n_ development in propylene polymerization by the LSF method using a TiCl_4_/1,3-diether/MgCl_2_-based catalyst; (**b**) An expanded view up to the polymerization time of 0.2 s [21].

With these modifications, the LSF system offers i) constant transfer over a wider range of the slurry concentration; ii) scalable polymerization, where the cumulated polymer yield is linearly correlated with the cumulated flow volume (Figure 4); and iii) precise polymerization experiments over a wider range of the polymerization time. Figure 5 represents typical results of propylene polymerization in the range of 0.03 to 2.5 s. It was found that the polymer yield increased linearly with the time up to 2.5 s (Figure 5a), where the yields were highly reproducible, and the corresponding time-yield curve linearly crossed the origin. These observations correspond to the fact that the number and nature/states of the active sites remained unchanged within the employed polymerization period (i.e., constant [C*] and *k*_p_), as well as the instantaneous mixing and activation of the catalyst at the T junction. Further, the linear increase of the yield up to 2.5 s assures that the flow rate of the polymerization slurry was hardly affected by the increase of viscosity in the course of the polymerization. The quasi-living nature of the polymerization was also confirmed by the linear increase of the *M*_n_ up to 0.2 s (Figure 5b). The *M*_n_ values gradually converged (Figure 5a) in the later stage of the polymerization (from 0.2 to 2.5 s) due to the enlarged contribution of chain transfer reactions. Based on the linear fit of the *M*_n_ development in the polymerization period of 0–0.2 s (Figure 5b), the average value of *k*_p_ was determined as 8.0 × 10^3^ L/mol·s, which was consistent with the value obtained for the same catalyst in the conventional SF polymerization [29]. The expansion of the polymerization range up to 2.5 s allowed to determine the average value of *k*_tr_ based on the linear fit of *M*_0_/*M*_n_ against 1/*t* (cf. Equation (2)), which was found to be 10 s^−1^. Thus, the LSF technique enables us to elucidate the olefin polymerization kinetics in accordance with the conventional SF technique, but at the same time the polymerization scalability and flow stability are successfully improved.

## 3. Chemical and Physical Transformations of Ziegler–Natta Catalysts over Quasi-Living Regime

The olefin polymerization rate per Ti (*R*_p_), which is also termed as the polymerization activity, can be represented by the following empirical equation [32,40],

(4) $$R_{p}(t)=-\frac{d[M(t)]}{dt}=k_{p}(t)[C^{*}(t)][M(t)]$$

with the first-order assumption for the monomer concentration. The symbols in Equation (4) have their usual meaning (cf. Equation (1),(2)). Although Equation (4) appears to be simple, it includes great complexities. This is because the polymerization kinetics are dictated by a series of complicated chemical and physical phenomena. The chemical phenomena include the formation of active sites after the reaction of supported TiCl_4_ with an alkylaluminum, the transformation of active sites, and the deactivation of active sites plausibly driven by agglomeration and/or over-reduction with the alkylaluminum [11,41,42]. On the other hand, the fragmentation of catalyst particles induced by polymer formation in the most-accessible pores leads to the exposure of hidden Ti species present on the surfaces of inner pores [29,43]. Diffusion resistance by the produced polymer also leads to a concentration gradient in the polymer/catalyst particles [44]. Thus, the parameters *k*_p_, [C*], and [M] in Equation (4) vary during polymerization due to these parallel chemical and physical phenomena, which necessitate time-resolved polymerization in order to identify the effect of individual phenomena on the polymerization kinetics.

The influence of the physical factors on the polymerization kinetics depends on the time scale of the polymerization, as well as the porosity and morphology of catalyst particles. For instance, the first fragmentation occurs only after obtaining a few g-polymer/g-catalyst [37], while the time scale of the chemical transformations roughly corresponds to the lifetime of one active site. The importance of the morphology of catalyst particles can be apprehended from the kinetic profiles of co-ground and chemically activated ZN catalysts. Co-ground catalysts mainly possess particles with irregular morphology and a smaller pore volume, in which the Ti species are concentrated on the topmost surfaces of the particles [29]. Thus, co-ground catalysts are generally featured with a high initial activity followed by a rapid decay of the polymerization rate [42]. On the contrary, chemically activated ZN catalysts with spherical particles possess build-up-type kinetics in propylene polymerization, which is attributed to gradual fragmentation and subsequent exposure of hidden Ti species uniformly distributed throughout the particles [10,29].

The SF polymerization had been applied for the time scale in which a steady-state active site concentration can be assumed for understanding the chemical transformations in ZN propylene polymerization. For example, Mori et al. found that the lifetime of the active sites on a TiCl_4_/dibutylphthalate/Mg(OEt)_2_-based ZN catalyst was significantly extended by ageing the catalyst with an alkylaluminum before the SF polymerization, plausibly due to the formation of active sites with higher stereospecificity as a result of interaction of TiCl_4_ with the alkylaluminum [45]. Combining a three-vessel SF system with TREF polymer analyses, Nitta et al. studied the successive formation, deactivation and transformation of stereospecific active sites on the same type of the catalyst by reacting the catalyst with an alkylaluminum for different durations, prior to the polymerization [31]. They found that i) the formation of active sites had an induction period in propylene polymerization with the employed catalyst, ii) the active sites with lower stereospecificity gradually deactivated or transformed into active sites with the highest stereospecificity, plausibly via interactions with the alkylaluminum, and iii) the fraction of aspecific active sites gradually increased, plausibly due to the abstraction of the internal donor by the alkylaluminum. On the other hand, many studies attempted to correlate the polymerization kinetics with physical phenomena, such as the morphological development of polymer/catalyst particles and diffusion of reagents. For example, Nooijen reported that diffusion of an alkylaluminum through the catalyst particles is critical for controlling fragmentation and the activity profile of a catalyst [46,47]. Tait et al. studied propylene polymerization using a silica-supported ZN catalyst. They reported that diffusion of the monomer and alkylaluminum deep inside the catalyst particles governed the polymerization kinetics [48]. Thus, understanding of the ZN olefin polymerization kinetics requires both of the chemical and physical viewpoints, whereas their cooperation in determining the kinetics was hardly investigated.

For this purpose, the initial stage of propylene polymerization using a TiCl_4_/diisobutylphthalate (DIBP)/Mg(OEt)_2_-based ZN catalyst was investigated by the LSF technique [39]. It is widely recognized that the initial stage of the polymerization, especially in the first few seconds, has a great influence on the polymerization kinetics, where the degree of catalyst activation and the first fragmentation of catalyst particles are the crucial factors for the subsequent kinetics [49,50]. The LSF technique allowed us to perform propylene polymerization in the range of 0.1 to 6 s, in which the degree of catalyst activation was altered by the alkylaluminum (triisobutylaluminum) concentration. In order to understand the influence of physical transformations on the initial polymerization kinetics, the living morphology development of the polymer/catalyst particles was tracked by scanning electron microscopy (SEM). The chemical transformations during polymerization, such as the variation in the nature of the active sites at different alkylaluminum concentrations, were identified by applying cross fraction chromatography (CFC) on the obtained polymers.

**Figure 6:**
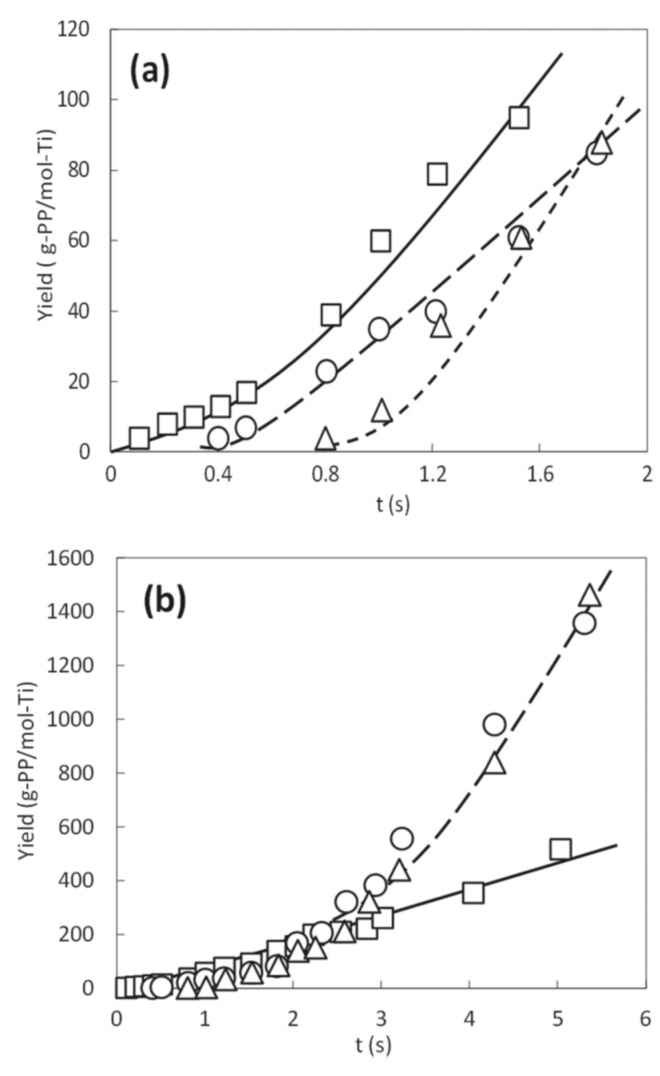
Propylene polymerization kinetics of a TiCl_4_/diisobutylphthalate/Mg(OEt)_2_-based catalyst at triisobutylaluminum concentrations of (Δ) 17.5 × 10^−3^ M, (o) 35 × 10^−3^ M, and (□) 70 × 10^−3^ M: (**a**) Up to ca. 2 s; (**b**) Up to ca. 6 s [39].

Figure 6a represents the kinetic profiles of LSF propylene polymerization in the range of 0.1 to 2 s, where the activation of the catalyst was found to be affected by the alkylaluminum concentration. At [Al] = 70 × 10^−3^ M, the polymer yield increased linearly with the time up to 0.5 s through the origin, suggesting the instantaneous activation of the catalyst at the T junction, as well as a steady state in propylene polymerization, which were in accordance with the conventional SF polymerization results using co-ground catalysts. In contrast, at a lower alkylaluminum concentration, there was an induction period in the time-yield curve, accompanied by a lower yield. The induction period in the initiation of the kinetic profiles was longer for [Al] = 17 × 10^−3^ M than for [Al] = 35 × 10^−3^ M. Nevertheless, the polymer yield developed proportionally to time even at the lower alkylaluminum concentrations in the beginning.

Figure 6b shows the complete kinetic profiles up to 6 s. At [Al] = 70 × 10^−3^ M, the linear increment of the polymer yield (up to 0.5 s) later accompanied a gradual non-linear growth, where the activity of the catalyst, represented by the gradient of the yield, increased monotonically with the time. This non-linear region in the kinetic profile corresponds to the build-up part of the polymerization kinetics of Mg(OEt)_2_-based ZN catalysts. Most importantly, the kinetic profile clearly demonstrated for the first time the transition of constant activity regime in the typical SF polymerization to build-up activity regime in the nominal polymerization. Similar trends were observed for the kinetic profiles at the lower alkylaluminum concentrations, where the non-linear growth started from ca. 0.8 s for [Al] = 35 × 10^–3^ M, and from ca. 1.2 s for [Al] = 17 × 10^–3^ M. Another interesting finding in Figure 6b was that a higher alkylaluminum concentration led to a higher yield in the linear region, whereas the yield became greater in the build-up region for the lower alkylaluminum concentrations due to greater curvatures of the time-yield curves. Thus, the alkylaluminum concentration was found to be a critical factor not only for the initiation but also for the build-up region of the kinetic profiles.

**Figure 7:**
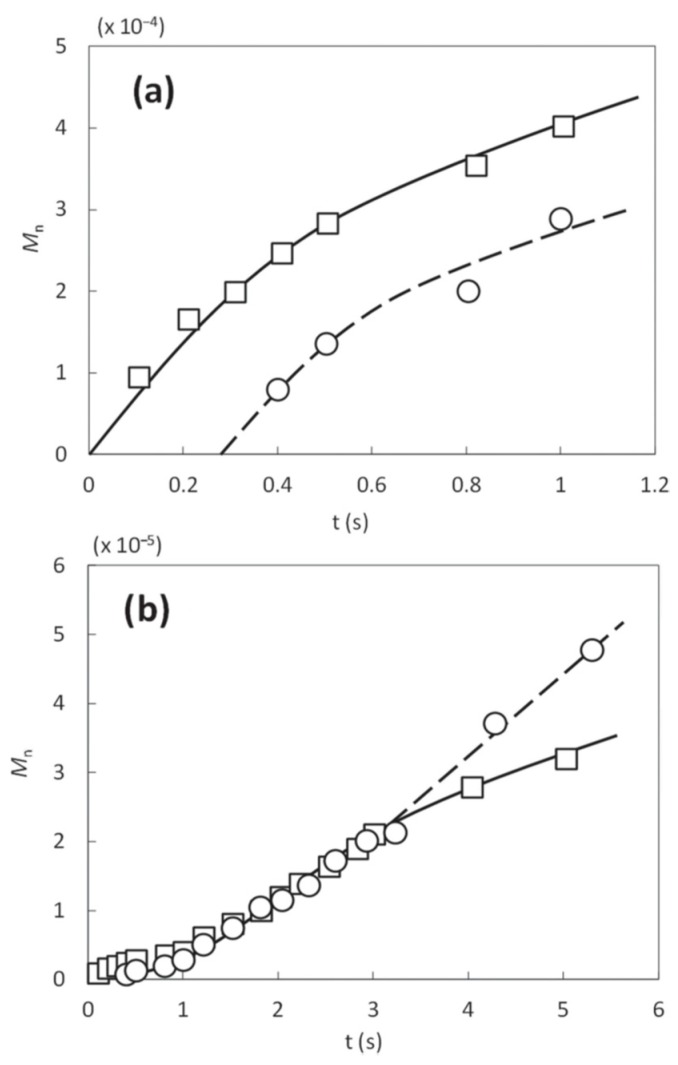
*M*_n_ development in propylene polymerization at triisobutylaluminum concentrations of (o) 35 × 10^−3^ M, and (□) 70 × 10^−3^ M: (**a**) Up to ca. 1.2 s; (**b**) Up to ca. 6 s [39].

**Figure 8:**
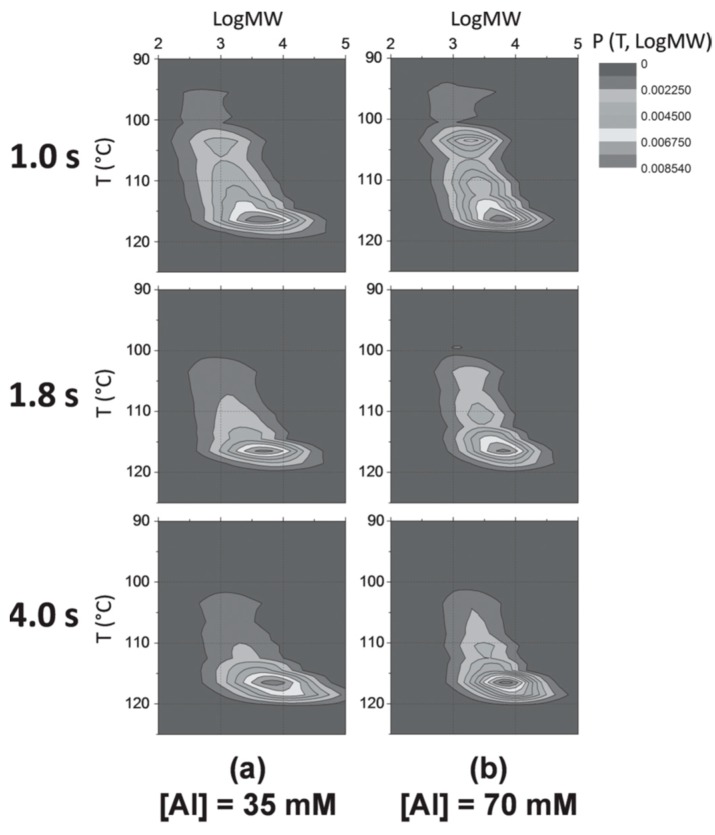
Cross fraction chromatography (CFC) contour plots of polymers obtained from LSF propylene polymerization at: (**a**) [Al] = 35 × 10^−3^ M; (**b**) [Al] = 70 × 10^−3^ M [39].

The dependence of the *M*_n_ on the polymerization time at two different alkylaluminum concentrations was investigated, which is shown in Figure 7. The *M*_n_ displayed a stepwise growth behavior along the time, which can be roughly divided into three regimes: i) a linear increment up to 0.4 s at [Al] = 70 × 10^–3^ M and up to 0.6 s at [Al] = 35 × 10^–3^ M due to quasi-living polymerization; ii) a converging behavior from 0.4/0.6 s up to 1.0 s due to a growing contribution of chain transfer reactions; and iii) a second linear increment from 1.0 s up to 3.0 s at [Al] = 70 × 10^–3^ M and up to 5.0 s at [Al] = 35 × 10^–3^ M with a greater slope i.e., an enhanced *k*_p_. The difference in the duration of the second linear *M*_n_ increment between the two alkylaluminum concentrations was plausibly attributed to the frequency of chain transfer to alkylaluminum. The regime iii) plausibly resulted from the delayed formation of the active sites with higher *k*_p_ (higher stereospecificity) and/or gradual transformation of the active sites with lower *k*_p_ (lower stereospecificity) into the active sites with higher *k*_p_, by interaction/reaction with the alkylaluminum [31]. Such formation/transformation of active sites in the course of the polymerization at different alkylalumium concentrations was studied by CFC (Figure 8), which is a 2D analysis between polymer molecular weight (MW) and tacticity distribution. Since the microstructures of polymers represent the average nature of the active sites on a catalyst, any change in the shape and position of the CFC contour plots reflects the variation in nature of the active sites. In Figure 8a ([Al] = 35 × 10^–3^ M), on increasing the polymerization time from 1.0 s to 1.8 s, the polymer fraction distribution shifted towards higher T (i.e., the stereoregularity of PP improved) and higher LogMW directions by disappearance of the tail of the contour concentrated at lower T and lower LogMW. This suggested that the average nature of the active sites became more isospecific, which accompanied a faster chain propagation. On further increase of the polymerization time up to 4.0 s, the tail of the contour further suppressed, while its breadth elongated in the direction of higher LogMW, plausibly due to an enhanced *k*_p_, as was explained for Figure 7. Similar contour plots were observed for [Al] = 70 × 10^–3^ M (Figure 8b), which suggested that the nature of the active sites is less affected by the alkylaluminum concentration. This was consistent with the fact that the *M*_n_ values of the obtained PP at both of the alkylaluminum concentrations were similar (Figure 7), except over 3.0 s.

From Equation (4), the observed transition from linear to build-up kinetics in Figure 6b could be attributed to both/either a gradual increase in [C*] and/or *k*_p_. In order to comprehend the origin of the kinetic transition, the *M*_n_ development at different alkylaluminum concentrations was compared with the yield development. It was found that the yield increased in a non-linear manner after the transition, while the *M*_n_ always developed linearly (except the converging step). Moreover, the *M*_n_ development was hardly affected by the alkylaluminum concentration, whereas the yield development strongly depended on it. The above facts suggest that the origin of the kinetic transition was less likely attributed to the enhanced *k*_p_ (i.e., chemical transformation), and more likely relevant to an enhanced [C*] driven by the gradual exposure of hidden Ti species with the progress of catalyst fragmentation (i.e., physical transformation) in the course of the polymerization.

**Figure 9:**
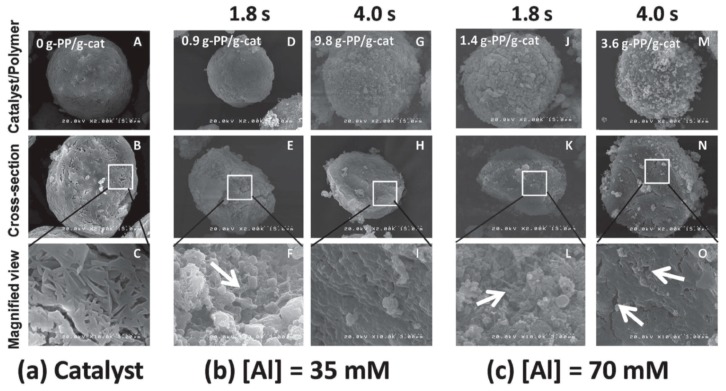
Scanning electron microscopy (SEM) images of surface and bulk morphology of: (**a**) A catalyst particle; (b) Polymer/catalyst particles obtained from LSF propylene polymerization at [Al] = 35 × 10^−3^ M; (c) Polymer/catalyst particles obtained from LSF propylene polymerization at [Al] = 70 × 10^−3^ M [39].

For a deeper understanding of the role of physical transformations in controlling the polymerization kinetics, the polymer/catalyst morphology development was tracked by SEM (Figure 9). For this, the polymerization was quenched by CO_2_ dissolved in cold heptane at −65 °C. Use of cold heptane decelerates, and CO_2_ terminates the polymerization, while the morphology of the polymer/catalyst particles remains intact after the quenching [36]. Figure 9a shows SEM images of the employed catalyst in terms of the particle surface and bulk morphology. A catalyst macroparticle had a nearly spherical shape, typical for Mg(OEt)_2_-based ZN catalysts [5,6]. The cross-sectional images revealed a layered structure of the macroparticle comprised of irregularly arranged lamellae as the secondary building units, where the interspaces among them were mainly identified as macropores. Figure 9b,c show SEM images of the polymer/catalyst particles produced in the LSF polymerization at the polymerization time of 1.8 s and 4 s, and at two different alkylaluminum concentrations. At the lower alkylaluminum concentration ([Al] = 35 × 10^−3^ M), the surface morphology of a polymer/catalyst particle obtained at 1.8 s was similar to that of a pristine catalyst particle, while its bulk morphology was changed, plausibly due to partial macropore filling by the formed polymer. In addition, some polymer fibrils were observed, suggesting the separation of stacked lamellae during fragmentation of the catalyst particles. The progress of the polymerization from 1.8 to 4.0 s led to an increase in the polymer yield from 0.9 to 9.8 g-PP/g-catalyst. As a result, the surface morphology of the polymer/catalyst particle became rougher with the formed polymer, and its bulk morphology became more compact, indicating that a major fraction of the macropores was filled with the polymer. At the higher alkylaluminum concentration ([Al] = 70 × 10^−3^ M), a polymer/catalyst particle obtained at 1.8 s exhibited a rough surface morphology, while the bulk morphology in terms of partial macropore filling and fibrillar polymer structures was similar, compared with the lower activator concentration. On increasing the polymerization time from 1.8 to 4 s, the outer surfaces of the polymer/catalyst particle became rougher again with a thick layer of formed polymer, whereas a large fraction of the macropores inside the particle remained unfilled. This fact suggests that at the higher alkylaluminum concentration, the polymer was preferentially formed on the outer surfaces, causing a lesser extent of fragmentation inside the catalyst particles, contrary to the results obtained for the lower alkylaluminum concentration.

Thus, the morphological difference of the polymer/catalyst particles between different alkylaluminum concentrations must be directly related to the observed difference in the kinetic profiles in Figure 6b. In general, the diffusion of an alkylaluminum deep inside the catalyst particles is recognized as a crucial factor for the fragmentation behavior at an early stage of the polymerization, where deeper diffusion results in more uniform fragmentation of the particles. At the higher alkylaluminum concentration ([Al] = 70 × 10^–3^ M), the degree of initial activation of the catalyst was higher, which facilitated the formation of a greater number of active sites on the outer surfaces of the catalyst particles, leading to the higher initial activity. However, the higher polymer yield in the beginning resulted in the formation of a thick polymer layer covering the macroparticles, and consequently retarded the diffusion of monomer and alkylaluminum inside the particles during the progress of the polymerization. In contrast, at the lower alkylaluminum concentration (e.g., [Al] = 35 × 10^–3^ M), the number of active sites formed in the beginning must be smaller, resulting in the lower initial activity. When the polymerization progressed, the mass transfer limitation of reagents was supposedly smaller due to a smaller amount of initially formed polymer, which facilitated the exposure of a greater number of hidden Ti species through uniform fragmentation of the catalyst particles.

From this kinetic study with the LSF technique, it was found that the catalyst experienced both physical and chemical transformations in the initial stage of the polymerization. However, catalyst fragmentation, i.e., physical transformation, was found to be mainly responsible for the build-up-type kinetics of propylene polymerization, whose origin can be attributed to the time-dependent increase in the active site concentration. The degree of activation of the catalyst (i.e., the alkylaluminum concentration) significantly regulated the polymerization kinetics, which is related to the diffusion limitation of reagents by the initially formed polymer on the surfaces of the catalyst macroparticles.

## 4. Large-Scale Stopped-Flow Technique with New Quenching Method

**Figure 10:**
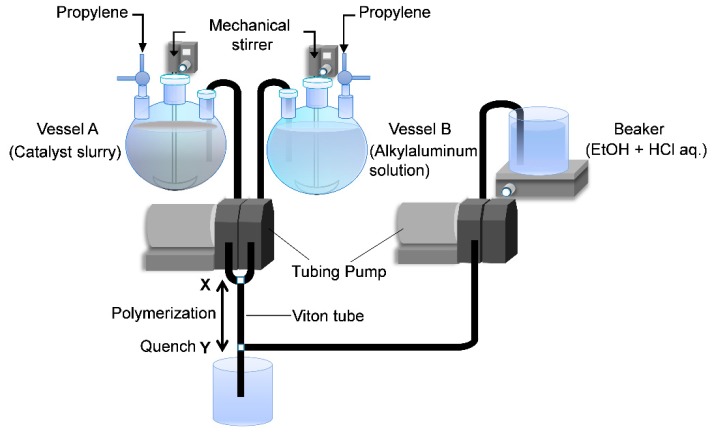
LSF apparatus with a new quenching method [38].

The LSF technique has successfully circumvented the limitation of poor polymer yields in conventional SF polymerization. It offers stable polymerization without the viscosity-induced flow rate reduction that is caused by polymer formation inside the tube. Ideally, the LSF system enables any arbitrary volume of slurry transfer to obtain a desired amount of polymer for extensive analyses. However, when the transferred volume of the polymerization slurry is increased, the volume of the quenching solution must be simultaneously increased for efficient quenching. This in turn lowers the efficacy of the homogenizer used for quenching beyond a certain volume limit. In order to further enhance the scalability of the LSF polymerization, a new quenching method was introduced [38], in which the quenching solution is pumped out from a third vessel through a Viton tube and allowed to contact the polymerization slurry at a second T junction. This three-vessel LSF apparatus with the new quenching method is demonstrated in Figure 10.

**Figure 11:**
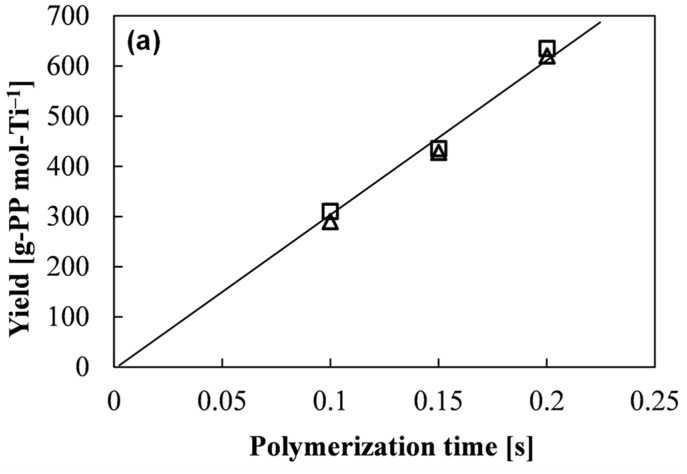

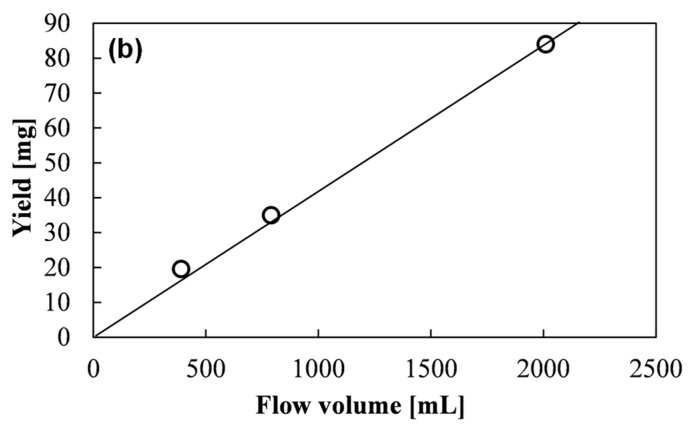
(**a**) Yield development in LSF propylene polymerization using a co-ground catalyst (TiCl_4_/1,3-diether/MgCl_2_): (Δ) New quenching and (□) conventional quenching; (**b**) Scalability test with the new quenching method. The tube length was fixed at 2 s, while the cumulated flow volume was increased [38].

In the SF polymerization, the instantaneous activation at the time scale of 0.01 s is facilitated by the catalyst particles, which act as micro-stirrers to endow instantaneous mixing between the catalyst slurry and activator solution at the T junction. The same principle was applied to the new quenching method, where the polymer/catalyst particles as micro-stirrers enable instantaneous quenching of the polymerization at the second T junction. The concept of the new quenching method was validated based on: i) the identical kinetic profiles using the original and the new quenching methods (Figure 11a); and ii) the improved scalability of the LSF polymerization using the new quenching method. In Figure 11b, it is shown that the LSF polymerization was linearly scaled up to 2000 mL of the polymerization slurry by employing the new quenching method, whereas the original method can quench at maximum 800–1000 mL of the slurry.

## 5. Regiochemistry of Ziegler–Natta Propylene Polymerization

**Scheme 1:**
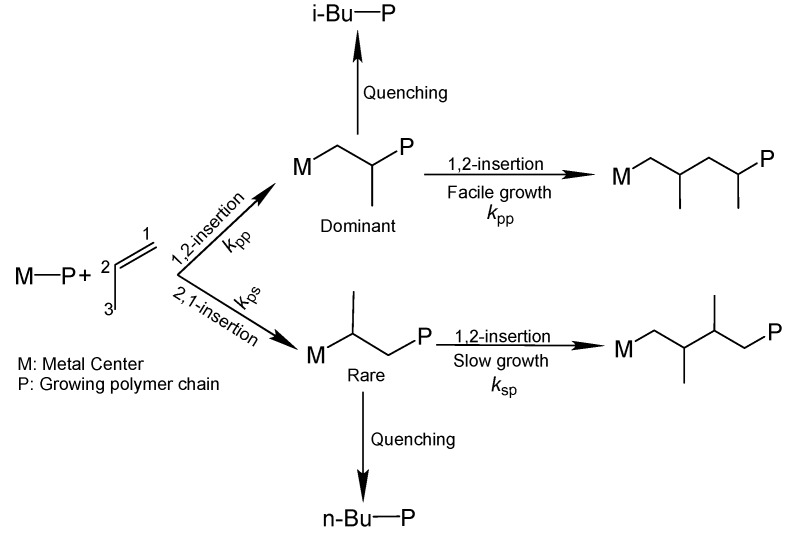
Regiochemistry of Ziegler–Natta (ZN) propylene polymerization.

The isoselective polymerization of propylene by heterogeneous ZN catalysts involves multiple cis-insertions of the prochiral monomer with the same enantioface, in which the stereoselection mechanism is mainly determined by the asymmetric nature of the active sites [51]. Propylene polymerization by ZN catalysts is not only highly stereoselective but also regioselective in favor of the primary (1,2) insertion of an incoming monomer. Yet, like stereo-misinsertion, the polymerization is also accompanied with occasional secondary (2,1) insertions of the monomer [52], leading to regiodefects in the growing polymer chain (Scheme 1). Although the content of regiodefects is estimated to be 0.01–0.1%, the occurrence of a regio-misinsertion is believed to slow down a subsequent monomer insertion by a factor of 10^2^–10^3^, as it introduces high steric hindrance at the metal center [53]. As a result, the propagating site after a regio-misinsertion of propylene becomes more or less dormant for further polymerization. This situation may lead to an accumulation of dormant sites in the system, which can be reactivated with small H_2_ molecules by a chain transfer mechanism [54,55]. Hence, the regiochemistry is considered to play a significant role on the polymerization kinetics as well as hydrogen response. Accordingly, the evaluation of both the regioselectivity of the propylene insertion and the dormancy of a regiodefected site is crucial to figure out the olefin polymerization kinetics.

The olefin polymerization kinetics, with the consideration of regiochemistry, is reasonably given by the first-order Markovian,

(5) $$Y=M_{0}\left(k_{pp}\left[C_{p}^{*}\right]+\;k_{ps}\left[C_{p}^{*}\right]+\;k_{sp}\left[C_{s}^{*}\right]+\;k_{ss}\left[C_{s}^{*}\right]\right)\left[M\right]t$$

(6) $$k\left[C^{*}\right]=k_{pp}\left[C_{p}^{*}\right]+k_{ps}\left[C_{p}^{*}\right]+k_{sp}\left[C_{s}^{*}\right]+k_{ss}\left[C_{s}^{*}\right]$$

(7) $$\left[C^{*}]=\left[C_{p}^{*}\right]+[C_{s}^{*}\right]$$

(8) $$\frac{d\left[C_{s}^{*}\right]}{dt}=k_{ps}\left[C_{p}^{*}]\left[M\right]-k_{sp}[C_{s}^{*}\right]\left[M\right]$$

where *k* stands for the apparent propagation rate constant, and the subscripts, p and s, describe the primary and secondary insertions, respectively. [C_p_*] and [C_s_*] correspond to the concentrations of 1,2- and 2,1-ended active sites. The rate constants, for example, *k*_ps_ corresponds to the rate constant of a secondary insertion after a primary insertion (Scheme 1), and so on. The other symbols have their usual meaning (cf. Equation (1),(2)). There are two limiting cases for Equation (8), which are described below:

Linear regime: When the 2,1-ended active sites are rare (i.e., [C_p_*] >> [C_s_*]) and almost linearly accumulated by occasional regio-misinsertions at an early stage of polymerization,

(9) $$[C_{s}^{*}]=k_{ps}[C_{p}^{*}]\left[M\right]t$$

Steady-state regime: When the generation of the 2,1-ended active sites is balanced by the recovery with the subsequent 1,2 insertion,

(10) $$k_{sp}[C_{s}^{*}]=k_{ps}[C_{p}^{*}]$$

Based on the kinetic parameters of Equation (5), the regioselectivity of the propylene insertion and dormancy of a regiodefected site can be expressed by the following equations,

(11) $$Regioselectivity\;=\;\frac{k_{pp}}{k_{pp}+\;k_{ps}}\;\times 100\;(\mathrm{linear}\;\mathrm{regime})$$

(12) $$Regioselectivity\;=\;\frac{k_{pp}\;[C_{p}^{*}]+\;k_{sp}\;[C_{s}^{*}]\;}{k\;[C^{*}]}\;\times 100\;(\mathrm{steady}-\mathrm{state}\;\mathrm{regime})$$

(13) $$Dormancy=\;\frac{k_{pp}}{k_{sp}}$$

Microstructural characterization of a polymer by ^13^C NMR spectroscopy can provide a deeper understanding of the polymerization mechanism. For instance, characterization of the primary structure of PP enables us to elucidate the stereoselection mechanism by applying a statistical model to the stereosequences in the main chain [56]. In this regard, it is also possible to identify the modes of monomer insertion (1,2 or 2,1 insertion) and estimate their relative rates (*k*_ps_/*k*_pp_). However, an extremely low concentration of 2,1 propylene units usually makes it difficult for the ^13^C NMR technique to estimate the amount of regioirregular enchainments in the polymer chain. To solve this issue, Busico et al. employed ^13^C-labeled PP samples prepared using less regioselective (e.g., C_2_-symmetric ansa-zirconocene) catalysts, which successfully enhanced the sensitivity of ^13^C NMR analysis with up to 50 times increment in signal-to-noise ratio for the important peaks [53]. As a result, they effectively estimated the percentage of 2,1-inserted propylene units internal to the main chain, and thus the ratio *k*_ps_/*k*_pp_. It was also confirmed that the sequential 2,1 units was very rare; about 5% of the total population of 2,1 units. Although informative, these findings were not sufficient to estimate the regioselectivity and dormancy of the employed catalysts.

Thus, the complete propylene polymerization kinetics can be obtained by evaluation of the eight unknown kinetic parameters [C*], [C_p_*], [C_s_*], *k*, *k*_pp_, *k*_ps_, *k*_sp_, *k*_ss_. However, this is practically impossible for usual propylene polymerization methods, especially with heterogeneous ZN catalysts, mainly because the most primitive parameters [C*] and *k* are unknown during the polymerization. Owing to this fact, several approximation methods had been pursued to elucidate the regiochemistry of propylene polymerization. For example, Busico et al. performed hydrooligomerization of propylene under the assumption of stationary-state conditions using both single-site molecular and heterogeneous ZN catalysts, in which chain transfer to monomer and/or alkylalumium, as well as isomerization reactions were negligible [54,57]. They plotted the mole ratio (*Q*_pH_/*Q*_sH_) between isobutyl and n-butyl chain ends of low molecular weight propylene hydrooligomers obtained at different propylene concentrations against [(1 + *Q*_sH_/*Q*_pH_) × *P*_n_]^–1^, where *P*_n_ is the number average degree of polymerization. Based on a linear regression analysis, the best-fit values for *k*_ps_/*k*_pp_ and for the product (*k*_sp_/*k*_ps_) (*k*_pH_/*k*_sH_) were obtained. They also performed propylene copolymerization in the presence of a small amount of ^13^C-enriched ethylene and plotted the mole ratio (*Q*_pE_/*Q*_sE_) of ethylene units that were adjacent to a 1,2- or a 2,1-inserted propylene unit against the ethylene/propylene feed ratio [53,58,59]. From the obtained linear plot, the best-fit values for *k*_pE_/*k*_ps_ and for the product (*k*_sp_/*k*_ps_) (*k*_pE_/*k*_sE_) were acquired. Since the regiochemistry of propylene insertion is (mainly) dictated by steric effects, it was assumed that small H_2_ or ethylene molecules have almost similar reactivity to a 1,2- and a 2,1-ended active sites, *i.e. k*_pH_/*k*_sH_ and *k*_pE_/*k*_sE_ were regarded close to unity, and therefore the products (*k*_sp_/*k*_ps_) (*k*_pH_/*k*_sH_) and (*k*_sp_/*k*_ps_) (*k*_pE_/*k*_sE_) were approximated to *k*_sp_/*k*_ps_. Consequently, they estimated the fraction of dormant chains, which is given by (1 + *k*_sp_/*k*_ps_)^–1^, for comparing the dormancy of various catalysts. Even though these elegant research works provided a solid foundation for understanding the regiochemistry of propylene polymerization, direct evaluation of all the eight unknown kinetic parameters of Equation (6) has not been achieved.

It should be noted that *k* and [C*] can be directly obtained by the SF propylene polymerization (cf. Equation (1),(3)). Also, due to the negligible occurrence of chain transfer reactions in quasi-living SF polymerization, the [C_p_*] and [C_s_*] can be derived by the chain end analyses of living polymers that are obtained by the instantaneous termination.

(14) $$\frac{\left[C_{p}^{*}\right]}{\left[C_{s}^{*}\right]}=\frac{\left[i-Bu\right]}{\left[n-Bu\right]}$$

where [i-Bu] and [n-Bu] represent the fraction of isobutyl and n-butyl chain ends, respectively. As the occurrence of two sequential regio-misinsertions is too rare to be observed, *k*_ss_ is regarded as zero. The remaining three first-order rate constants are derived by the combination of Equation (6), (9) (or 10), and (15),

(15) $$\frac{\left[S\right]}{\left(\left[S\right]+\left[P\right]\right)}=\frac{k_{sp}\left[C_{s}^{*}\right]}{k\left[C^{*}\right]}$$

where [P] and [S] represent the fraction of 1,2 and 2,1 propylene units internal to the main chain. [i-Bu], [n-Bu], [P] and [S] are given by ^13^C NMR analyses of SF polymers.

In this regard, ultra-short-time propylene polymerization by the LSF technique with the new quenching method (Figure 10) is undisputedly the most direct way to determine the regioselectivity of propylene polymerization and the dormancy of a regiodefected site. This is because the polymerization kinetics below 0.1 s are supposedly well separated from chain transfer reactions. Thus, the extremely short polymer chains should contain a dominant fraction of chain ends. The microstructural analyses of these living polymers were performed with the aid of the state-of-the-art high-temperature (HT) ^13^C cryo-probe NMR in order to elucidate the kinetics of the regiochemistry in the first-order Markovian.

**Figure 12:**
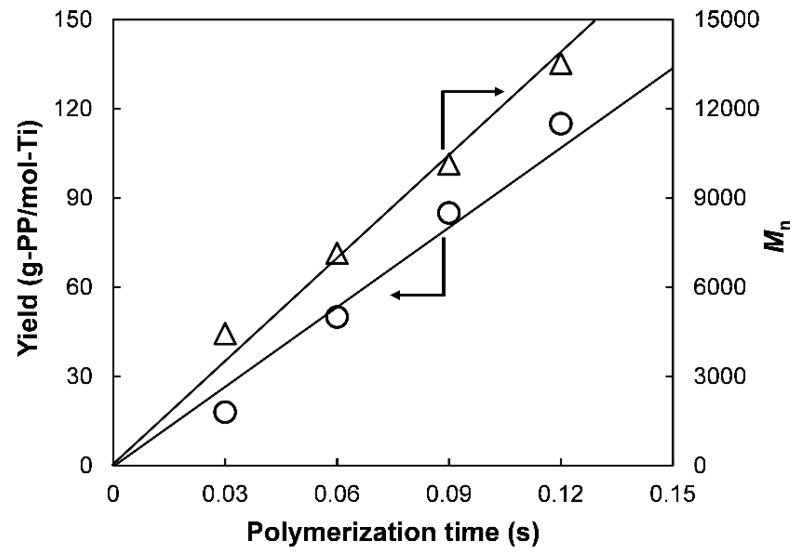
Yield and *M*_n_ development in short-time LSF propylene polymerization using a TiCl_4_/ethylbenzoate/MgCl_2_ co-ground catalyst.

Figure 12 represents the short-time propylene polymerization kinetics performed with the LSF technique using a TiCl_4_/ethylbenzoate (EB)/MgCl_2_ co-ground catalyst. The living nature was confirmed by the linear *M*_n_ development.

**Figure 13:**
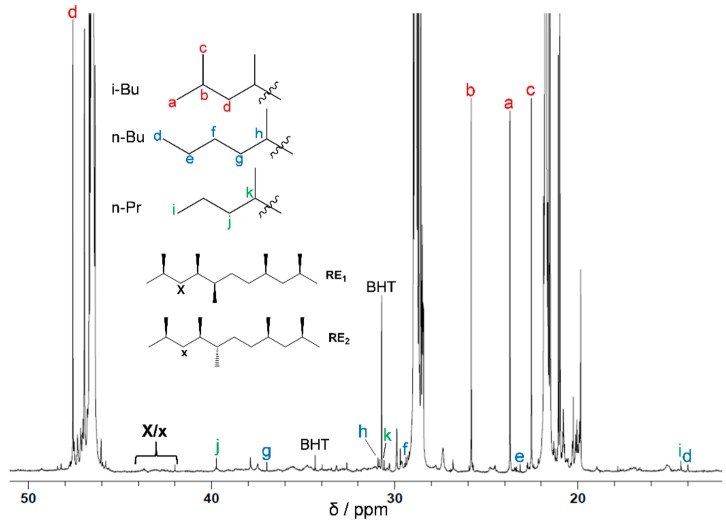
High-temperature (HT) ^13^C cryo-probe NMR spectrum of a polypropylene (PP) sample obtained from LSF propylene polymerization at 0.06 s. Minor peaks arose from regioirregular sequences and chain ends of PP.

Figure 13 represents the ^13^C NMR spectrum of a PP sample formed at 0.06 s, in which the major peaks correspond to the resonances of regioregular and isotactic sequences of the main chain. The peaks corresponding to the isobutyl unit are clearly observed. The isobutyl unit arose from chain initiation by triisobutylaluminum as well as chain termination by acidic quenching at the last 1,2-inserted propylene. Apart from these, many minor peaks were found, which were related to various kinds of chain ends (or chain heads) and to 2,1 propylene units internal to the main chain. Among these, the n-butyl chain end unit is ascribed to last 2,1-inserted propylene and the n-propyl unit corresponds to a chain head that arose from minor occurrence of chain transfer to monomer. The stereochemical arrangements of 2,1 regio-misinsertion in the main chain (RE_1_ and RE_2_) are also shown in the spectrum [60].

The stereoregularity of the produced polymers calculated through the mesopentad (*mmmm*) fraction was found to be ca. 90%, reasonable for the employed catalyst. The results of the ^13^C NMR chain end analyses are summarized in Table 1. On increasing the polymerization time from 0.06 to 0.12 s, the fraction of the isobutyl chain end unit was found to be almost halved. This fact indicated that the majority of the living chain end was the last 1,2-inserted propylene unit, whose fraction became half when the molecular weight was doubled from 0.06 to 0.12 s. In contrast, the fraction of the n-butyl chain end unit decreased roughly by 30% on increasing the polymerization time to 0.12 s, probably because of accumulation of the last 2,1-inserted propylene unit with certain accuracy limit. For main chain analyses, the peaks for the carbons **X** and **x** (Figure 13) adjacent to 2,1 propylene units were chosen, as they were well separated from other minor peaks. From the ^13^C NMR peak integration, the fraction of internal 2,1 propylene units was found to be very low (ca. 0.04 ± 0.01 mol%). Even though a large error range in ^13^C NMR analyses was originated by the scarcity of the regio-misinsertion, the polymerization kinetics given by the first-order Markovian was solved and the estimated kinetic parameters are listed in Table 2 and Table 3.

In Table 2, the apparent kinetic parameters *k* and [C*] were obtained as the best-fit values from the linear yield and *M*_n_ development against the polymerization time (Figure 12), in accordance with typical SF polymerization results using co-ground catalysts. According to an estimated large error range in the ^13^C NMR analyses, the first-order Markovian represented by Equations (5–15) was solved at each polymerization time and hence the kinetic parameters in Table 2 are given with a range. The fraction of dormant species (i.e., [C_s_*]) was found to occupy 6–7% among the living chain end. Estimation of the kinetic rate constants for different insertion reactions based on either the linear regime or the steady-state regime of regio-misinsertion revealed that the secondary (2,1) insertion of propylene is an (extremely) slow reaction, whose specific rate (*k*_ps_) is at least 2 × 10^3^ times lower than that of the primary insertion (*k*_pp_). The regioselectivity of the propylene insertion for the employed system was found to be high (>99.9%), suggesting that one out of 10^3^–10^4^ insertions were secondary insertions. Most importantly, the obtained kinetic rate constants in Table 2 enabled us to roughly estimate the dormancy (*k*_pp_/*k*_sp_) of a regiodefected site, which was found to be in the range of 160–200, in nice agreement with that estimated from propylene hydrooligomerization experiments using heterogeneous ZN catalysts [54].

In a recent article, Yu et al. studied the role of regiochemistry of propylene polymerization on the catalyst performance with the LSF technique in the range of 0.07–2.0 s using TiCl_4_/EB/MgCl_2_ and TiCl_4_/DIBP/MgCl_2_-based catalysts [61]. In the case of the EB-based catalyst, the reported regio-misinsertion of ca. 0.03 mol% was in close agreement with the value measured from Figure 13. In contrast to the EB-based catalyst, the DIBP-based catalyst was explained as highly dormant even in the LSF regime. They reported that at the very early stage (*t* ≤0.2 s), the fraction of the 1,2-ended active sites was higher than that of the 2,1-ended active sites. The fraction of the 2,1-ended active sites rapidly developed with the time and after 0.3 s they became greater than that of the 1,2-ended active sites. These results suggested that a large fraction of the active sites became dormant even at the time scale of less than 0.5 s. After 0.5 s, the linearly evolved dormant sites gradually reached a stationary state, in which the formation of the dormant sites was plausibly balanced by the reactivation with the subsequent 1,2 insertion. Another important finding in this research was that the stereoregularity of the obtained polymers gradually became lower and the molecular weight distribution became broader with the progress of the polymerization. These facts suggested that the most active and stereospecific active sites gradually became dormant with the progress of the polymerization. Thus, the results obtained in the research work of Yu et al. validated the inevitable role of molecular H_2_ in controlling the polymerization kinetics, which can suppress the poisoning of a catalyst in the course of the propylene polymerization.

## 6. Conclusions

The transient nature, heterogeneity, and scarce concentration of the active sites of heterogeneous Ziegler–Natta (ZN) catalysts prevent their spectroscopic characterizations, which prompted to apply the stopped-flow (SF) technique in a quasi-living polymerization stage for obtaining a direct correlation between polymer microstructures and the nature of the active sites. Over the last two decades the technique has been extensively applied to clarify several aspects of the olefin polymerization mechanisms using ZN catalysts. However, the small amount of obtained polymers in a short time had always been the major problem of the SF technique in extensive polymer analyses. To circumvent this limitation, a large-scale SF (LSF) system was introduced, which relies on the identical principle of the conventional SF system, but with greatly improved scalability to obtain a sufficient amount of polymers and polymerization stability over a wider range of the polymerization time i.e., from a few tens of milliseconds to several seconds The efficacy of the LSF technique can be exploited to uncover several unknown issues in ZN catalysis such as the role of physical and chemical transformations of a ZN catalyst on the polymerization kinetics at an early stage in the range of 0.1 to 6 s. It was found that a time-dependent increase in the active site concentration due to catalyst fragmentation was responsible for the build-up-type kinetics of propylene polymerization. Also, the degree of activation of the catalyst was found to significantly influence the polymerization kinetics, which could be attributed to the diffusion limitation of reagents by the initially formed polymer on the surfaces of the catalyst macroparticles. The LSF system was further equipped with a new quenching method, which can effectively quench any desired volume of polymerization slurry to obtain a sufficient amount of polymers even at the time scale of less than 0.1 s. With this modification, the LSF technique was successfully applied to access the propylene polymerization kinetics at a very early stage below 0.1 s to investigate the dormant processes in propylene polymerization. Most importantly, the microstructural analyses of the living polymers with the aid of the state-of-the-art high-temperature ^13^C cryo-probe NMR enabled to kinetically elucidate the regiochemistry of propylene polymerization based on the first-order Markovian statistics. This review article thus describes the development and application of the LSF technique for elucidation of the fundamental aspects of industrial ZN catalysis and is expected to be useful for researchers in dealing with complicated catalyst systems.

## Figures and Tables

**Table 1:** ^13^C NMR chain end analysis of PP obtained from LSF propylene polymerization.

Carbon	Mole Fraction ^a^		Chain End Contribution (mol%)	
	0.06 s	0.12 s	0.06 s	0.12 s
Methine carbon of main chain	100	100	–	–
‘a’ of i-Bu unit	0.96	0.54	87.1 ^b^	86.2 ^b^
‘d’ of n-Bu unit	0.03 ± 0.01	0.02 ± 0.01	6.0 ± 2	6.9 ± 3.4
‘i’ of n-Pr unit	0.035 ± 0.005	0.02 ± 0.005	6.9 ± 1	6.9 ± 1.7

^a^ Calculated by NMR peak integration. ^b^ Estimated as {[i-Bu (total)] – [n-Bu] – [n-Pr]}/2.

**Table 2:** Estimation of kinetic parameters based on the first-order Markovian statistics ^a^.

k (L/mol·s)	[C*] (mol%)	[C_p_*] (mol%)	[C_s_*] (mol%)	Linear Approximation			Steady-State Approximation		
				k_pp_ (L/mol·s)	k_sp_ (L/mol·s)	k_ps_ (L/mol·s)	k_pp_ (L/mol·s)	k_sp_ (L/mol·s)	k_ps_ (L/mol·s)
4.3 × 10^3^	0.99	0.92–0.93	0.06–0.07	4.6–4.7 × 10^3^	23.4–27.6	1.1–1.9	4.6–4.7 × 10^3^	23.4–27.6	1.8–1.9

^a^ Estimated based on Equations (1,3,5–10,14,15).

**Table 3:** Estimation of the regioselectivity of propylene insertion and dormancy of a regiodefected site ^a.^

Regioselectivity (%)		Dormancy	
Linear Approximation	Steady-State Approximation	Linear Approximation	Steady-State Approximation
>99.9	>99.9	167.5	199.9

^a^ Estimated based on the values of the kinetic rate constants obtained from Table 2 and using Equations (11–13).
